# Lemon Oils Attenuate the Pathogenicity of *Pseudomonas aeruginosa* by Quorum Sensing Inhibition

**DOI:** 10.3390/molecules26102863

**Published:** 2021-05-12

**Authors:** María Constanza Luciardi, María Amparo Blázquez, María Rosa Alberto, Elena Cartagena, Mario Eduardo Arena

**Affiliations:** 1 INBIOFAL (Institute of Pharmaceutical and Food Biotechnology), CONICET NOA Sur, Avenida Kirchner 1900, Tucumán 4000, Argentina; cotiluciardi@hotmail.com (M.C.L.); maria.alberto@fbqf.unt.edu.ar (M.R.A.); elena.cartagena@fbqf.unt.edu.ar (E.C.); 2 Facultad de Bioquímica, Química y Farmacia, Universidad Nacional de Tucumán (UNT), Ayacucho 471, Tucumán 4000, Argentina; 3 Departament de Farmacologia, Facultat de Farmàcia, Universitat de València, Avd. Vicent Andrés Estellés s/n, 46100 Burjasot, Valencia, Spain

**Keywords:** *Citrus* peel, biofilm, quorum sensing, motility, elastase, pyocyanin, virulence factors

## Abstract

The chemical composition of three *Citrus limon* oils: lemon essential oil (LEO), lemon terpenes (LT) and lemon essence (LE), and their influence in the virulence factors production and motility (swarming and swimming) of two *Pseudomonas aeruginosa* strains (ATCC 27853 and a multidrug-resistant HT5) were investigated. The main compound, limonene, was also tested in biological assays. Eighty-four compounds, accounting for a relative peak area of 99.23%, 98.58% and 99.64%, were identified by GC/MS. Limonene (59–60%), *γ*-terpinene (10–11%) and *β*-pinene (7–15%) were the main compounds. All lemon oils inhibited specific biofilm production and bacterial metabolic activities into biofilm in a dose-dependent manner (20–65%, in the range of 0.1–4 mg mL^−1^) of both strains. Besides, all samples inhibited about 50% of the elastase activity at 0.1 mg mL^−1^. Pyocyanin biosynthesis decreases until 64% (0.1–4 mg mL^−1^) for both strains. Swarming motility of *P. aeruginosa* ATCC 27853 was completely inhibited by 2 mg mL^−1^ of lemon oils. Furthermore, a decrease (29–55%, 0.1–4 mg mL^−1^) in the synthesis of Quorum sensing (QS) signals was observed. The oils showed higher biological activities than limonene. Hence, their ability to control the biofilm of *P. aeruginosa* and reduce the production of virulence factors regulated by QS makes lemon oils good candidates to be applied as preservatives in the food processing industry.

## 1. Introduction

*Citrus*, one of the most popular world fruit crops, contains active phytochemicals like essential oils, polyphenols, vitamins, dietary fibers, minerals and carotenoids that can protect health [1]. After oranges and mandarins, lemon is considered a major citrus fruit and is commercially cultivated worldwide due to its medicinal importance [2].

The fruit peels, the main waste obtained from citrus juice production, are a potential source of secondary plant metabolites and essential oils associated with a broad range of health benefits [1]. These valuable by-products could be sources of preservatives and functional compounds and can be used to develop innovative food products that are safe and with health-promoting activities [3]. The *Citrus* peel essential oils commercial production is commonly done by hydrodistillation and cold pressing the *Citrus* peels [4].

*Citrus* essential oils (CEOs) have been used for a long time in traditional medicine. Their properties, such as antioxidant, antibacterial, antiviral, fungicidal and anti-inflammatory activities, have been widely demonstrated [5,6]. It is known that the biological activities of the essential oils depend on their chemical constituents and in turn, these depend on geographical and environmental conditions (temperature, rainfall, altitude and hours of sunshine) [2,7].

The volatile and semi-volatile compounds represent between 85 and 99% of the CEOs and are generally characterized by more than 200 compounds, many of them of interest to the food and perfume industries [8]. By being generally recognized as safe (GRAS) by the Food and Drug Administration (FDA), they have achieved increased acceptance in the food industry, where they are commonly used as preservatives, flavorings and antioxidants [4,7,9].

The main CEO components are classified into terpenes (monoterpenes and sesquiterpenes), alcohols, aldehydes and esters [4]. In *Citrus limon* essential oils, the most abundant monoterpene hydrocarbons reported are d-limonene, *γ*-terpinene and *β*-pinene and between oxygenated monoterpenes, the most abundant are *α*-terpineol, nerol and geraniol [10]. Quantitatively, limonene is the main compound of *C. limon* essential oils at levels typically ranging between 70 and 48% [8].

Lemon essential oils (LEOs) have antibacterial and fungicidal properties on foodborne pathogens and spoilage bacteria [5,9,11,12]. Moreover, they showed considerable antioxidant and antimicrobial properties in a food model [12]. All of this suggests its potential to be used as a natural preservative to prevent product contamination.

The antimicrobial resistance problem has not escaped the food industry since various bacteria have become resistant to commonly employed food preservatives. Microbial biofilms have led to significant economic impacts in many food industries like dairy, fish processing, poultry, meat and Ready-To-Eat [13]. Biofilm and several virulence factors within the biofilms allow the spread and persistence of foodborne pathogenic bacteria in the food processing industries’ equipment and surfaces. The bacterial performance within biofilms is controlled by the phenomenon known as Quorum sensing (QS), where bacteria produce chemical signals and express virulence genes in a cell population density-dependent manner [14]. In the food industry, there are many environmental factors such as substratum, food matrix composition, temperature, hydrodynamic effects, oxygen concentration and microbial interactions that favor bacterial biofilms’ formation and development [15]. Essential oils have proved to be potent candidates to reduce or control biofilms associated with foodborne pathogens [5].

*Pseudomonas aeruginosa*, a pathogenic and spoilage bacterium commonly found in the environment and food processing facilities, has a great tendency to form biofilms [16]. *P. aeruginosa* biofilm adheres to various biotic and abiotic surfaces and it is tough to eradicate in food processing industries. Biofilm formation on foods and industrial food equipment is a serious problem causing food spoilage and the emergence of foodborne diseases [13]. Since the biofilm is a survival mechanism, but it also contributes to virulence and persistence, it has been suggested that preventing its development is a way of addressing the biofilm problem in the food industry. *Pseudomonas* spp. (including *P. aeruginosa*) is one of the most frequently isolated bacteria from spoiled seafood, chill-stored foods, milk, meat products and water used for food processing and due their proteolytic and lipolytic activities contribute to the losses in food quality and safety [16,17,18]. For these reasons, finding effective antimicrobial agents against bacterial biofilms has become a challenge for researchers worldwide.

We previously demonstrated that two CEOs (mandarin and grapefruit essential oils) inhibit the *P. aeruginosa* biofilm establishment and other virulence factors controlled by QS [19,20]. This work is aimed to study the chemical composition of three commercial oils obtained from *C. limon* (L.) Burm. f. (Rutaceae) (lemon essential oil, lemon terpenes and lemon essence), the inhibitory potential on virulence factors (biofilm, elastase, pyocyanin and swarming and swimming motilities) and the production of QS signals by two *P. aeruginosa* strains.

## 2. Results

### 2.1. Chemical Composition

After GC/MS analysis, a total of eighty-four volatile organic compounds were evidenced in the commercial lemon oils. LT and LE showed greater variability of compounds (sixty-nine and sixty-seven constituents) than LEO (forty-six constituents). Components were grouped in homologous series of monoterpene hydrocarbons, oxygenated monoterpenes, sesquiterpene hydrocarbons, oxygenated sesquiterpenes and alkanes, aldehydes/ketones and esters and then listed according to Kovats retention index calculated in GC on an apolar HP-5MS column (Table 1).

High amounts of monoterpene hydrocarbons were identified in LEO, LT and LE (91.41, 91.37 and 87.34%, respectively), being limonene the main constituent (59.14, 59.28 and 60.07%; respectively), followed by *β*-pinene (15.41, 14.2 and 6.68%, respectively) and *γ*-terpinene (10.48, 11.05 and 11.57%, respectively). The other compounds in this group were found in amounts less than 1%, except for myrcene, *α*-pinene and sabinene.

The oxygenated monoterpenes represented 5.07, 4.84 and 8.17% of the total compounds in the LEO, LT and LE, respectively and showed qualitative and quantitative differences between the three samples. In general, LE had the highest quantity and variety of oxygenated monoterpenes, among which the most abundant compound was terpinen 4-ol, which was found in much lower values in the LEO and LT. Among the hydrocarbon sesquiterpenes, only bicyclogermacre and *β*-bisabolene reached 1% in LE. Finally, the oxygenated sesquiterpenes germacrene d-4-ol, spathulenol and caryophyllene oxide were detected only in LT.

### 2.2. Virulence Factors Inhibition

#### 2.2.1. Biofilm

**Figure 1:**
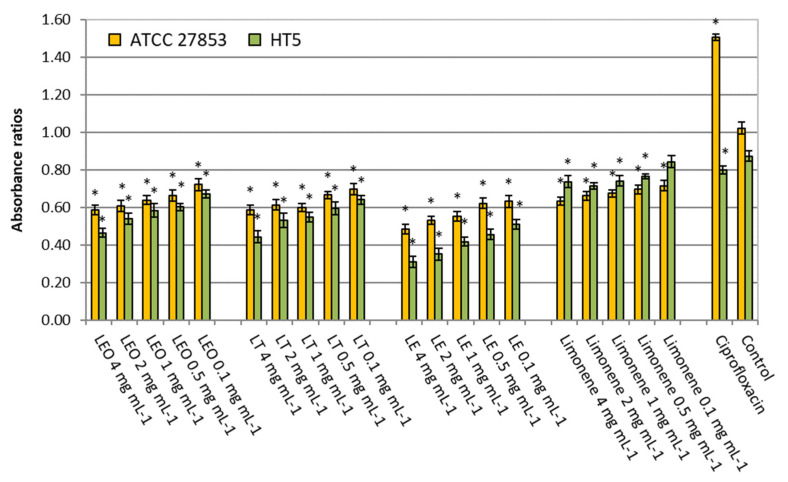
Specific biofilm formed by *Pseudomonas aeruginosa* strains without (control) and in the presence of ciprofloxacin (5 µg mL^−1^) or different lemon oils concentrations (0.1–4 mg mL^−1^). LEO: Lemon Essential Oil, LF: Lemon Terpenes, LE: Lemon Essence. Asterisk indicates significant differences compared to the respective control (Tukey’s multiple range test, *p* < 0.05).

All essential oil samples were able to inhibit the specific biofilm production of the two strains tested, ATCC 27853 and HT5 (Figure 1). The reducing effect was in a dose-dependent manner. LEO inhibited 29–43% the biofilm of the ATCC 27853 strain and 23–47% of the HT5 strain, in the range of concentrations tested (0.1–4 mg mL^−1^). With LT, the inhibitions were 32–43% for ATCC 27853 and 27–49% for HT5. LE was the most effective in reducing the biofilm formation of both strains of *P. aeruginosa* (38–53% for ATCC 27853 and 42–65% for HT5). However, for the major compound limonene, little or no inhibition was observed, especially with the strain resistant to multiple antibiotics (30–38% for ATCC 27853 and 3–16% for HT5). These results suggest a possible synergistic interaction between the constituents of lemon oils that improve the limonene bioactivity.

**Figure 2:**
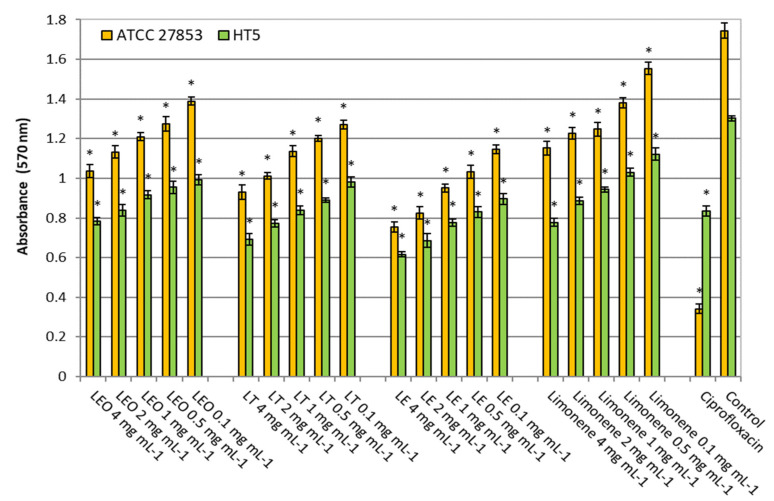
Metabolic activity of *Pseudomonas aeruginosa* strains into biofilm without (control) and in the presence of ciprofloxacin (5 µg mL^−1^) or different lemon oils concentrations (0.1–4 mg mL^−1^). LEO: Lemon Essential Oil, LF: Lemon Terpenes, LE: Lemon Essence. Asterisk indicates significant differences compared to the respective control (Tukey’s multiple range test, *p* < 0.05).

Similarly, all lemon oils effectively inhibited bacterial metabolism in biofilms in a dose-dependent manner (Figure 2). For the ATCC 27853 strain, the inhibitions in the ranges tested (4–0.1 mg mL^−1^) were 20–40%, 27–47%, 34–57% and 11–34% for LEO, LT, LE and limonene, respectively. While for the HT5 strain the inhibitions were 24–40%, 25–47%, 31–53% and 14–40%, respectively. Once again, LE was the most active sample, probably due to the higher proportion of oxygenated and esterified monoterpenes compared to the other oils.

#### 2.2.2. Elastase

**Figure 3:**
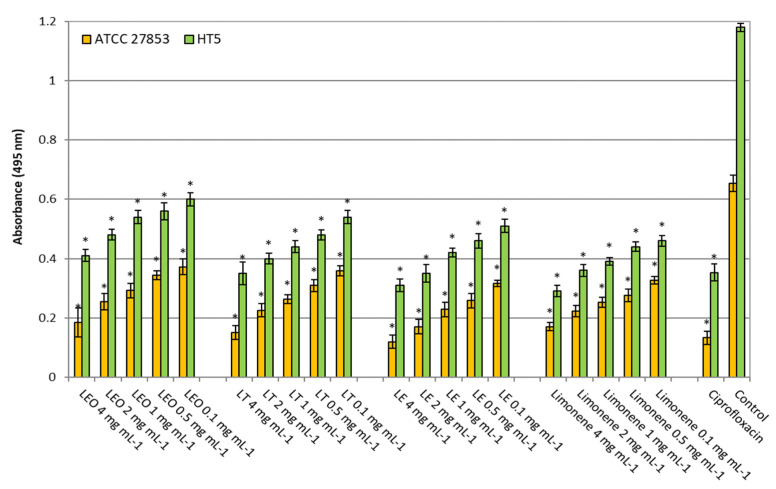
Elastase activity of *Pseudomonas aeruginosa* strains without (control) and in the presence of ciprofloxacin (5 µg mL^−1^) or different lemon oils concentrations (0.1–4 mg mL^−1^). LEO: Lemon Essential Oil, LF: Lemon Terpenes, LE: Lemon Essence. Asterisk indicates significant differences compared to the respective control (Tukey’s multiple range test, *p* < 0.05).

The three lemon oils strongly reduced (in a dose-dependent manner) the elastase activity of *P. aeruginosa* ATCC 27853 and HT5 to the same extent as limonene (Figure 3). In *P. aeruginosa* ATCC 27853, an inhibition of 43–72%, 45–77%, 51–82% and 50–74% was determined, in the presence of LEO, LT, LE and limonene, respectively, in a concentration range of 0.1–4 mg mL^−1^. In *P. aeruginosa* HT5, the decreases were 49–65%, 54–70%, 57–74% and 61–75% in the presence of LEO, LT, LE and limonene, respectively. It is essential to highlight that all the samples at 0.1 mg mL^−1^ inhibited the enzymatic activity of both strains tested by around 50%. 

#### 2.2.3. Pyocyanin

The commercial lemon oils inhibited the formation of the toxic pigment pyocyanin (Table 2). In *P. aeruginosa* ATCC 27853, after exposure to LEO, LT, LE and limonene, the decrease in pyocyanin were 39–61%, 43–63%, 45–62% and 24–58%, respectively, for the concentrations ranged of 0.1–4 mg mL^−1^. Similar results were observed in *P. aeruginosa* HT5. In the presence of LEO, LT, LE and limonene, the pigment formation decreased 34–62%, 28–64%, 36–62% and 20–60%, respectively, in the range of 0.1–4 mg mL^−1^.

#### 2.2.4. Bacterial Motility

Commercial lemon oils and their main constituent, limonene, moderately inhibited motility in a liquid medium, known as swimming (individual movement), in both *P. aeruginosa* strains. In the ATCC 27853 strain, LEO, LT, LE and limonene inhibited the bacterial motility by 45, 31, 50 and 50%, at the highest concentration tested. For the strain HT5, the swimming reductions were 33, 28, 28 and 31% for LEO, LT, LE and limonene, respectively (Table 3).

The lemon oils inhibited more than 50% of the bacterial group movement (swarm) of *P. aeruginosa* ATCC 27853 in all the concentrations tested. However, the total inhibition was reached at 2 mg mL^−1^ for all lemon oils. At the lowest concentration tested (0.1 mg mL^−1^), the inhibitions were 71, 64 and 57% in the presence of LEO, LT and LE, respectively. The anti-swarm effect against *P. aeruginosa* HT5 was also significant. In this case, the higher inhibitory effect was reached at 4 mg mL^−1^, with reductions of 69, 78, 56 and 54% by LEO, LT, LE and limonene, respectively (Table 4).

#### 2.2.5. Autoinducer Production

**Figure 4:**
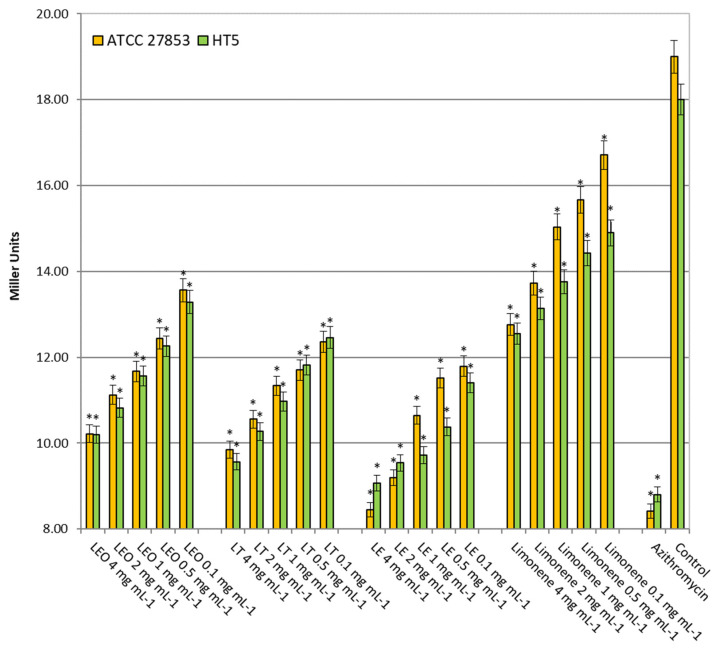
*β*-galactosidase activity of *Pseudomonas aeruginosa* strains without (control) and in the presence of azithromycin (5 µg mL^−1^) or different lemon oils concentrations (0.1–4 mg mL^−1^). LEO: Lemon Essential Oil, LF: Lemon Terpenes, LE: Lemon Essence. Asterisk indicates significant differences compared to the respective control (Tukey’s multiple range test, *p* < 0.05).

Figure 4 shows the autoinducer production by lemon oils. In *P. aeruginosa* ATCC 27853, inhibitions of 29–46%, 35–48%, 38–55% and 12–33% were recorded in the presence of LEO, LT, LE and limonene, respectively, for the concentration range of 0.1–4 mg mL^−1^. Similar results were obtained in the *P. aeruginosa* HT5 strain with inhibitions in the AHL production of 26–43%, 31–47%, 37–50% and 17–30%, respectively.

## 3. Discussion

Foodborne diseases increasingly attract public attention worldwide because they can cause significant losses in society and considerably affect the economy [21]. The antimicrobial resistance issue has not spared the food industry; for example, *P. aeruginosa* acquired resistance to synthetic food preservatives commonly employed in foodstuff [22].

In this sense, it is known that essential oils and their constituents prolong food stability during storage by inhibiting the growth of pathogenic or spoilage microorganisms [23]. The present study explores the antivirulence activities of lemon oils and their major component limonene against *P. aeruginosa* strains.

The analysis of the volatile profile of the three lemon oils shows that they mainly contain monoterpene, with limonene, *β*-pinene and *γ*-terpinene as the major compounds. These results were comparable to the characteristic compositions reported in the literature. In LEO from Turkey and India, limonene was the main constituent, with values of 78.93 and 53.57%, respectively, followed by *β*-pinene (5.08 and 7.44%) [2]. However, in another work, D-limonene, *p*-cymene, *β*-pinene were found in percentages of 52.85%, 14.36% and 13.69%, respectively [9]. According to the general results, limonene was also the dominant compound in Argentina, United States and Spain LEOs; however, the second main compound was *γ*-terpinene (8.8, 10.5% and 9.66%) and *β*-pinene the following important compound [11,24]. Nevertheless, Iran’s LEO D-limonene (46.93%) was followed by *γ*-terpinene (16.89%), tri-cyclen (6.67%), 1-*β*-pinene (4.69) and 2-*β*-pinene (3.86%) [12]. The bibliographic search reveals that there are not yet many works on the essential oil of *C. limon* variety limoneira, which was studied in this work. It is known that the chemical composition depends on the variety, growing regions, storage times, growing seasons, which in turn, is specific to the geographical area and this could influence the antibiofilm activity [25,26].

The growth of *P. aeruginosa* was only weakly inhibited (18–26%) at the higher concentrations assayed (2 and 4 mg mL^−1^) (Figure S1, Supplementary Materials), while the virulence factors production was reduced since the lowest concentration used (0.1 mg mL^−1^). For this reason, the lemon oils could be considered antipathogenic (non-microbicidal virulence factors inhibitors), with the advantage that they would not produce bacterial resistance and represent a subtler method of infectious control [27].

Commercial lemon oils (LEO, LT and LE) significantly reduced the production of AHLs and, subsequently, biofilm formation and other virulence factors as elastase, pyocyanin and swarming motility, important indicators of QS operon in *P. aeruginosa*. This QS system controls bacterial communication associated with various cellular activities like the invasion of niches, defense systems, mobilizing and biofilm formation to survive against hostile environments [14].

Since it was recently shown that 90% of *Pseudomonas* spp. isolated from food are biofilm producers [18], it is crucial to find compounds that inhibit their virulence. Some studies have demonstrated the antibiofilm ability of *Citrus changshan-huyou* [28] and *Citrus medica* [26,29] against *Listeria monocytogenes* and *Staphylococcus aureus* biofilm. It has been said that *P. aeruginosa* biofilm is more challenging to control than *L. monocytogenes* and *S. aureus* biofilm because the outer membrane of the Gram-negative bacteria could act as a barrier that prevents CEO penetration [26]. However, in line with the present results, *Citrus paradisi* (grapefruit) and *Citrus reticulata* (mandarin) essential oils were able to inhibit *P. aeruginosa* biofilm formation and the biofilm metabolic activity [19,20].

With respect to bacterial metabolic activity into biofilm, the samples studied were able to inhibit it. Contrary to our results, *Citrus medica* essential oil increases the metabolic activity of *S. aureus* cells in the biofilm in response to the stress suffered by exposure to the essential oil [26] and grapefruit essential oil has limited or no capacity to inhibit *P. aeruginosa* metabolic activity in biofilm [30]. Besides biofilm, extracellular virulence factors secretion also plays a vital role in the effective pathogenesis of *P. aeruginosa*. It was previously reported that some essential oils could interfere with the QS system regulation and the formation of virulence factors [31,32]. In line with this, the autoinducer production decreased between 26 and 55% in the lemon essential oils assayed; this result is similar to that found in grapefruit and mandarin essential oils [19,20]. Moreover, it was informed that *Citrus* extracts also decreased autoinducer production by about 90%, measured by a bioluminescence assay using *Vibrio harveyi* [33]. However, another LEO did not inhibit the production of violacein by *C. violaceum*, indicating that they do not interfere with the QS system [31].

The lower inhibition of QS by limonene is well correlated with a 20% inhibition of long-chain AHL production, without inhibiting C4-AHL synthesis, at 0.1 mg mL^−1^ as previously reported [34].

The diminution of QS autoinducer is well correlated with the inhibition of several virulence factors controlled by QS, such as elastase and pyocyanin. Elastase, a hydrolytic enzyme that breaks the host tissue by cleavage of extracellular matrix, was present in food-associated *P. aeruginosa* [17]. Further, the pigment pyocyanin interferes with numerous cellular functions and plays a significant role in human infections. In fact, *Pseudomonas* spp. strains isolated from food are good pigment producers [18]. 

In the present work, the elastase activity was inhibited between 43 and 82% by the lemon essential oils in. These results are lower than those observed for mandarin essential oil and similar to those found in grapefruit essential oil, tested at the same concentrations as this work [19,20].

Concerning the motility controlled by QS, in concordance with our results, hydroalcoholic extracts (96% ethanol) from *Citrus* peels, including *C. limon*, *C. medica* and *C. aurantium*, reduced *Campylobacter jejuni* swarm motility (35–59%) [32]. 

The commercial lemon oils tested also inhibited the swimming motility of *P. aeruginosa*, which QS does not regulate. This motility plays a fundamental role in the first stage of biofilm formation in cell/cell and cell/surface adhesion. Therefore, by inhibiting this movement, the formation of the bacterial biofilm would be avoided and for this fact, the inhibition of swimming in *P. aeruginosa* would contribute to lemon oils being powerful anti-biofilm agents.

The present work demonstrated that the pure limonene compound has inhibitory effects on the production of virulence factors. In general, this effect was less effective than those observed in lemon oils. These results are consistent with previous studies reporting antimicrobial effects of essential oils due to a complex interaction between its different components, even those present in low quantities [35,36].

In silico computational studies, Arjmandi et al. [37] suggested that several components found in lemon essential oils could diminish the *P. aeruginosa* biofilm formation by interacting with three key proteins involved in QS.

Likewise, in vitro, several LEO individual components inhibited biofilm formation, swarming, swimming and twitching motilities of two important bacteria causing vegetable spoilage, *Pseudomonas fluorescens* and *Erwinia carotovora* [38].

Interestingly, terpinen-4-ol significantly reduced swarming (33.3%) and swimming (25%) motilities patterns, as well as elastase activity (50%) and pyocyanin production (33%) in *P. aeruginosa* [39]. This compound is present in higher percentages in LE than in LEO and LT, which is correlated with the higher anti QS activity of LE. 

According to our research, lemon oils have a great potential to be used as a natural food preservative. The reductions observed in QS-dependent virulence factors (biofilm, elastase, swarming and formation of the pigment pyocyanin) would be due to a decrease in the synthesis of AHL-type QS signals, which leads us to argue that these lemon oils interfere in the bacterial communication system, which would attenuate the pathogenicity of *P. aeruginosa*. Further research should be carried out to explore their applicability in the food industry.

## 4. Materials and Methods

### 4.1. Samples

Three commercial lemon oils provided by ‘‘Citrusvil’’ Company (San Miguel de Tucumán, Argentina) were studied. The sample named *lemon essential oil* (LEO) was obtained by cold pressure of the fruit peels (99.9% of the commercial oil produced industrially). The sample named *lemon terpenes* (LT) was collected by steam distillation of the liquid discharged from the cold pressing oil centrifuge that did not separate in the initial centrifugation process. The sample named *lemon essence* (LE) was obtained by steam distillation of the oil recovered during making concentrated lemon juice. The samples were stored in the dark at 4 °C and dissolved in DMSO/Water (1:1) for biological assays. Limonene (the main compound found in the samples) from Sigma-Aldrich (Buenos Aires, Argentina) (purity ≥ 96.5%) was also processed in biological analyses.

### 4.2. Gas Chromatography-Mass Spectrometry

Gas chromatography-mass spectrometry (GC-MS) analysis was carried out with 5973N Agilent equipment (Agilent, Valencia, Spain) with a capillary column (95% dimethylpolysiloxane–5% diphenyl), HP-5M (30 m long and 0.25 mm i.d. with 0.25 mm film thickness), according to Luciardi et al. [19]. The column temperature program was 60 °C for 5 min, with 3 °C min^−1^ increases to 180 °C, then 20 °C min^−1^ increases to 280 °C, which was maintained for 10 min. The carrier gas was helium at a flow rate of 1 mL min^−1^. Split mode injection (ratio 1:30) was employed. Mass spectra were taken over the *m*/*z* range of 30–650 with an ionizing voltage of 70 eV. The compounds identification was based on corresponding their mass spectra peaks with those found in NIST 11 Mass Spectral Library. The Kovats retention indices (RIs) calculated using co-chromatographed standard hydrocarbons relative to C8–C30 *n*-alkanes) were compared with those from the literature [40] and they were used as a supplementary tool to support MS findings. The quantification was obtained from the peak area percent reports without correction factors, using the normalization method.

### 4.3. Strains and Growth Medium

Two strains were used; *P. aeruginosa* ATCC 27853 as a reference and *P. aeruginosa* HT5, a multi-antibiotic resistant isolated from a patient with food poisoning [19]. The strains were cultured at 37 °C in Luria–Bertani (LB) medium.

### 4.4. Bacterial Growth Measurement

In a microtiter plate, 180 µL of each strain suspension (OD 0.12 ± 0.01 at 560 nm) from an exponential phase culture, were mixed with 20 µL of each sample solution (1, 5, 10, 20 and 40 mg mL^−1^) to reach final concentrations of 0.1, 0.5, 1, 2 and 4 mg mL^-1^ in the wells (*n* = 8). As controls, vehicle (DMSO/water, 1:1) or ciprofloxacin (5 μg mL^−1^) were added instead of the sample. Non inoculated control wells with 180 µL culture media and 20 µL samples, or vehicle were also prepared (*n* = 8). After 24 h incubation at 37 °C, the growth was measured at 560 nm using a microplate reader (Power Wave XS2) Biotek, Winooski, Vermont, USA.

### 4.5. Biofilm Biomass Quantification Assay

Biofilm formation was quantified using the micro method proposed by O’Toole and Kolter [41]. The bacterial growth supernatants obtained as mentioned above were discarded and the biofilm fixed to the polystyrene was stained with crystal violet. The absorbance was measured at 540 nm in a microtiter plate reader (Power Wave XS2, Biotek). As controls, the vehicle or ciprofloxacin (5 µg mL^−1^) were added instead of the sample.

The specific biofilm formation index (BFI) was determined according to Teh et al. [42] by applying the formula:

BFI = (AB − CW)/G.(1)

AB is the OD_540nm_ of the stained (crystal violet) attached microorganisms, CW is the optical density of the stained non inoculated control wells and G is the OD_560nm_ of the bacterial growth.

This index correlates the biofilm formed with bacterial growth.

### 4.6. Biofilm Metabolic Activity Assay

The bacterial metabolic activity into biofilm was assessed using the 3-[4,5-dimethylthiazol-2-yl]-2,5-diphenyltetrazolium bromide (MTT) reduction assay with some modifications [19,43]. Two hundred microliters of each bacterial suspension (OD 560 nm, 0.09 ± 0.02) from an exponential phase culture were inoculated in the wells of 96-well microtiter plates and incubated for 24 h at 37 °C. After incubation, bacterial cultures were removed and the microplates were air-dried. Subsequently, 180 µL of PBS (pH 6.5) and 20 µL of each sample solution (*n* = 8) were added per well (final concentrations of 0.1, 0.5, 1, 2 and 4 mg mL^−1^ in the wells). The plates were incubated at 37 °C for 24 h and after that, they were washed with PBS. Then 100 µL of MTT solution (0.5 mg mL^−1^) were dispensed into each well and incubated for 3 or 6 h at 37 °C. The insoluble purple formazan formed was dissolved in DMSO. Finally, the absorbance was measured at 570 nm using the microplate reader. As controls, the vehicle or ciprofloxacin (5 µg mL^−1^) were added instead of the sample.

### 4.7. Elastase B Activity and Pyocyanin Quantification

Each sample at 0.1, 0.5, 1, 2 and 4 mg mL^−1^ final concentration was added to a culture of each *P. aeruginosa* (ATCC 27853 or HT5) (OD560 = 0.1 ± 0.02) and was incubated at 37 °C overnight. In the supernatants, elastase enzymatic activity was assessed using an elastin-congo red reagent [44] and the pyocyanin concentration was established after extractions with chloroform and HCl (0.2 M) [45]. A positive control without lemon oils and a negative control without inoculation were prepared for all the assays. The procedures were performed in quintupled.

### 4.8. Swarming and Swimming Motility

The motility was assayed as was described previously [46]. Briefly, LB agar 0.5% (swarm media) and 0.3% (swim media) (wt/vol) at a temperature between 45–50 °C with different sample concentrations or vehicle were poured into Petri dishes. The plates were allowed to solidify at room temperature. Subsequently, *P. aeruginosa* (ATCC 27853 or HT5) broth culture (LB, 37 °C, 24 h) was inoculated into the center of each plaque, by sterile toothpick (swim) or by putting 2 μL aliquot (swarm). Bacteria spreading from the inoculation spot were measured after 24 h at 37 °C. Motility radio was measured with Image J software and compared with the vehicle (DMSO/water) control. 

### 4.9. Quantification of N-acyl Homoserine Lactones (AHLs)

To study the QS inhibition, AHLs were quantified by the *β*-galactosidase activity test using *P. aeruginosa* qsc 119, a strain unable to produce its own AHLs. The enzyme activity produced by the reporter strain in response to exogenous active signal molecules generated by wild-types *P. aeruginosa* (ATCC 27853 or HT5) is directly related to the concentration of autoinducers [47].

Cell-free culture supernatant was obtained from each wild-type cultivated during 24 h in the presence of the samples (0.1, 0.5, 1, 2 and 4 mg mL^−1^, final concentrations) (*n* = 5). The *β*-galactosidase activity was determined spectrophotometrically [48,49]. Positive control with the vehicle instead of the sample and a negative control without inoculation were prepared. Azithromycin (5 μg mL^−1^) was used as QS positive control.

## Figures and Tables

**Table 1:** Chemical constituents of essential oil, terpene fraction and essence from the fruit peels of *C. limon* (L.) Burm. f. (Rutaceae).

Compounds			Peak Area (%)		
	RI ^a^	KI ^b^	Lemon Essential Oil	Lemon Terpenes	Lemon Essence
**Monoterpene hydrocarbons**			**91.41**	**91.37**	**87.34**
*α*-thujene	932	930	0.35	0.34	0.21
*α*-pinene	940	939	1.64	2.35	1.99
Camphene	954	954	0.05	0.04	0.05
Sabinene	978	975	1.76	1.58	4.39
*β*-pinene	982	979	15.41	14.20	6.68
*β*-myrcene	995	990	1.65	1.49	1.32
*α*-terpinene	1016	1017	0.14	-	-
*p*-cymene	1024	1024	0.11	0.29	0.02
Allocimene	1131	1132	-	-	t
Limonene	1040	1029	59.14	59.28	60.07
*cis*-ocymene	1046	1037	0.06	0.04	0.15
*trans*-ocymene	1055	1050	0.10	0.08	-
*γ*-terpinene	1064	1059	10.48	11.05	11.57
Terpinolene	1092	1088	0.54	0.64	0.88
**Oxygenated monoterpenes**			**5.07**	**4.84**	**8.17**
*cis*-sabinene hydrate	1078	1070	0.09	0.10	-
*trans*-sabinene hydrate	1098	1098	0.07	-	-
Linalool	1102	1095	0.18	0.24	0.29
*endo*-fenchol	1114	1116	-	-	0.04
*cis-p*-menth-2-en-1-ol	1122	1121	-	-	0.04
*cis*-limonene oxide	1134	1136	-	0.02	0.01
*cis*-*p*-mentha-2,8-dien-1-ol	1136	1137	-	t	t
*trans*-limonene oxide	1139	1142	-	0.01	-
*trans-p*-menth-2-en-1-ol	1140	1140	-	-	t
Camphor	1144	1146	-	0.02	0.02
Camphene hydrate	1147	1149	-	-	0.01
Citronellal	1154	1153	0.10	0.13	-
Borneol	1167	1169	-	-	t
Terpinen-4-ol	1176	1177	0.05	0.11	1.89
*E*-isocitral	1183	1180	-	-	0.01
*α*-terpineol	1189	1188	0.39	0.38	1.45
Nerol	1230	1229	0.02	0.02	0.14
Neral	1240	1238	0.01	1.09	1.17
Carvone	1244	1243	1.16	0.01	t
Geraniol	1258	1252	-	-	0.07
Geranial	1275	1267	1.85	1.58	1.09
Isobornyl acetate	1284	1285	-	0.01	t
Methyl geranate	1321	1324	-	0.01	0.01
*trans*-carvyl acetate	1334	1342	-	-	t
Citronellyl acetate	1352	1352	0.03	0.04	0.07
Neryl acetate	1365	1361	0.79	0.75	1.22
Geranyl acetate	1382	1381	0.30	0.30	0.58
Limonen-10-yl-acetate	1406	1395	0.01	0.01	0.02
*p*-menth-1-en-9-yl acetate	1415	1423	-	-	t
Neryl propanoate	1451	1454	0.01	0.01	0.01
Geranyl propanoate	1471	1477	-	0.01	0.01
**Sesquiterpene hydrocarbons**			**2.28**	**1.79**	**3.63**
*δ*-elemene	1333	1338	-	t	t
α-cubebene	1345	1348	-	-	t
*α*-copaene	1372	1376	-	0.01	t
*β*-cubebene	1384	1388	-	t	-
*α*-*cis*-bergamotene	1410	1412	0.05	0.04	0.05
*β*-caryophyllene	1414	1419	0.35	0.29	0.37
*α-trans*-bergamotene	1433	1434	0.66	0.54	0.78
*cis-β*-farnesene	1438	1442	-	t	t
*α*-humulene	1447	1454	0.03	0.02	0.02
*trans-β*-farnesene	1453	1456	0.06	0.04	0.06
*β*-santalene	1455	1459	0.02	0.02	0.02
*γ*-curcumene	1474	1482	0.02	0.01	0.02
Valencene	1486	1496	-	0.03	0.14
Bicyclogermacrene	1489	1500	0.08	0.06	1.06
*trans*-α-bisabolene	1497	1507	0.08	0.06	0.11
*β*-bisabolene	1505	1505	0.93	0.65	1.06
*cis-γ*-bisabolene	1510	1515	0.01	0.01	0.02
*δ*-cadinene	1517	1523	-	0.01	0.01
*trans-γ*-bisabolene	1526	1531	-	t	0.01
*cis- α*-bisabolene	1537	1536	-	0.01	0.02
**Oxygenated sesquiterpenes**			**0.07**	**0.02**	**t**
Germacrene d-4-ol	1569	1575	-	t	-
Spathulenol	1572	1578	-	t	-
Caryophyllene oxide	1574	1583	-	0.01	-
*α*-bisabolol	1680	1685	0.07	0.01	-
*β*-bisabolenal	1759	1769	-	-	t
**Alkanes, aldehydes/ketones and esters**			**0.44**	**0.56**	**0.38**
6-methyl-5-hepten-2-one	990	985	-	0.02	-
Octanal	1005	998	0.13	0.13	-
Octanol	1079	1068	-	-	0.04
Nonanal	1106	1100	0.16	0.21	0.14
Dodecane	1197	1200	-	0.01	-
Decanal	1202	1201	0.08	0.11	0.12
Octanol acetate	1210	1213	-	0.01	-
Tridecane	1296	1300	-	t	0.01
Undecanal	1303	1306	0.04	0.04	0.05
Nonanyl acetate	1308	1311	-	t	0.01
Tetradecane	1395	1400	0.02	0.01	0.02
Dodecanal	1403	1408	0.01	0.01	-
Hexadecane	1592	1600	-	t	t
Tetradecanal	1604	1611	-	t	-
**Total VOCs**			**99.23**	**98.58**	**99.64**

^a^ RI, Retention Index relative to C8-C32 *n*-alkane on HP-5MS column, ^b^ KI, Kovats Retention Index. t: traces < 0.01, VOCs: Volatile Organic Compounds.

**Table 2:** Pyocyanin pigment production from *Pseudomonas aeruginosa* strains.

**Samples**	***P. aeruginosa* ATCC 27853**				***P. aeruginosa* HT5**			
Control	0.85 ± 0.04				0.74 ± 0.02			
**Concentration**	**LEO**	**LT**	**LE**	**Limonene**	**LEO**	**LT**	**LE**	**Limonene**
4 mg mL^−1^	0.33 ± 0.04 *	0.31 ± 0.03 *	0.32 ± 0.03 *	0.36 ± 0.02 *	0.28 ± 0.02 *	0.27 ± 0.02 *	0.28 ± 0.03 *	0.30 ± 0.02 *
2 mg mL^−1^	0.38 ± 0.1 *	0.36 ± 0.03 *	0.34 ± 002 *	0.44 ± 0.02 *	0.31 ± 0.04 *	0.29 ± 0,03 *	0.31 ± 0.04 *	0.38 ± 0.03 *
1 mg mL^−1^	0.41 ± 0.03 *	0.38 ± 0.03 *	0.37 ± 0.03 *	0.48 ± 0.02 *	0.34 ± 0.04 *	0.36 ± 0.03 *	0.34 ± 0.03 *	0.46 ± 0.03 *
0.5 mg mL^−1^	0.46 ± 0.03 *	0.42 ± 0.03 *	0.42 ± 0.02 *	0.54 ± 0.03 *	0.37 ± 0.02 *	0.45 ± 0.04 *	0.42 ± 0.05 *	0.53 ± 0.01 *
0.1 mg mL^−1^	0.52 ± 0.02 *	0.48 ± 0.02 *	0.47 ± 0.03 *	0.65 ± 0.02 *	0.49 ± 0.03 *	0.53 ± 0.02 *	0.47 ± 0.03 *	0.59 ± 0.03 *

LEO: Lemon Essential Oil, LT: Lemon Terpenes, LE: Lemon Essence. Data represent the mean absorbance ± SD (*n* = 5). Asterisks indicate that the sample shows significant differences compared to the respective control (Tukey’s multiple range test, *p* < 0.05).

**Table 3:** *Pseudomonas aeruginosa* swimming motility.

**Samples**	***P. aeruginosa* ATCC 27853**				***P. aeruginosa* HT5**			
Control	21.80 ± 0.03				25.20 ± 0.06			
**Concentration**	**LEO**	**LT**	**LE**	**Limonene**	**LEO**	**LT**	**LE**	**Limonene**
4 mg mL^−1^	12.00 ± 0.04 *	15.0 0 ± 0.04 *	11.00 ± 0.07 *	11.00 ± 0.04 *	17.00 ± 0.06 *	18.20 ± 0.03 *	18.10 ± 0.07 *	17.50 ± 0.05 *
2 mg mL^−1^	13.90 ± 0.06 *	15.70 ± 0.03 *	15.90 ± 0.06 *	14.20 ± 0.03 *	17.60 ± 0.02 *	18.60 ± 0.02 *	18.20 ± 0.03 *	18.10 ± 0.04 *
1 mg mL^−1^	14.90 ± 0.02 *	16.70 ± 0.03 *	16.80 ± 0.03 *	19.50 ± 0.04 *	18.20 ± 0.03 *	19.10 ± 0.02 *	18.90 ± 0.02 *	19.20 ± 0.03 *
0.5 mg mL^−1^	16.00 ± 0.04 *	19.00 ± 0.07 *	22.30 ± 0.04	22.20 ± 0.03	18.70 ± 0.03 *	25.10 ± 0.02	25.20 ± 0.06	25.40 ± 0.04
0.1 mg mL^−1^	16.60 ± 0.04 *	19.90 ± 0.02 *	22.60 ± 0.06	22.60 ± 0.02	19.20 ± 0.03 *	26.00 ± 0.02	26.10 ± 0.02	25.80 ± 0.03

LEO: Lemon Essential Oil, LT: Lemon Terpenes, LE: Lemon Essence. Data represent the mean absorbance ± SD (*n* = 5). Asterisks indicate that the sample shows significant differences compared to the respective control (Tukey’s multiple range test, *p* < 0.05).

**Table 4:** *Pseudomonas aeruginosa* swarming motility.

**Samples**	***P. aeruginosa* ATCC 27853**				***P. aeruginosa* HT5**			
Control	14.30 ± 0.05				13.00 ± 0.04			
**Concentration**	**LEO**	**LT**	**LE**	**Limonene**	**LEO**	**LT**	**LE**	**Limonene**
4 mg mL^−1^	ND	ND	ND	ND	4.00 ± 0.04 *	2.90 ± 0.02 *	5.70 ± 0.05 *	6.00 ± 0.04 *
2 mg mL^−1^	ND	ND	ND	2.30 ± 0.05 *	6.30 ± 0.05 *	6.50 ± 0.04 *	8.90 ± 0.02 *	7.00 ± 0.04 *
1 mg mL^−1^	2.90 ± 0.04 *	3.10 ± 0.06 *	4.20 ± 0.05 *	5.00 ± 0.04 *	7.80 ± 0.03 *	8.10 ± 0.06 *	9.50 ± 0.04 *	7.40 ± 0.04 *
0.5 mg mL^−1^	3.40 ± 0.04 *	4.20 ± 0.03 *	5.90 ± 0.04 *	9.20 ± 0.03 *	9.00 ± 0.04 *	8.90 ± 0.06 *	10.90 ± 0.08 *	8.90 ± 0.02 *
0.1 mg mL^−1^	4.20 ± 0.03 *	5.20 ± 0.03 *	6.10 ± 0.02 *	10.26 ± 0.05 *	11.0 ± 0.07 *	10.70 ± 0.05 *	11.00 ± 0.06 *	11.00 ± 0.07 *

LEO: Lemon Essential Oil, LT: Lemon Terpenes, LE: Lemon Essence. Data represent the mean radio (mm) ± SD (*n* = 5). Asterisks indicate that the sample shows significant differences compared to the respective control (Tukey’s multiple range test, *p* < 0.05). ND: No detectable.

## Data Availability

The data presented in this study are available in the article and the Supplementary Materials.

## Supplementary Materials

The following are available online, Figure S1: *Pseudomonas aeruginosa* growth without (control) and in the presence of ciprofloxacin (5 µg mL^−1^) or different lemon oils concentrations (0.1–4 mg mL^−1^). LEO: Lemon Essential Oil, LF: Lemon Terpenes, LE: Lemon Essence. Asterisk indicates significant differences compared to the respective control (Tukey’s multiple range test, *p* < 0.05).

## Sample Availability

Samples of the lemon oils are available from the authors and commercially (“Citrusvil’’ Company).
