# Supplementary material for: Lemon Oils Attenuate the Pathogenicity of Pseudomonas aeruginosa by Quorum Sensing Inhibition
Source: Molecules. 2021 May 12;26(10):2863. doi: 10.3390/molecules26102863 (PMC8151035; doi:10.3390/molecules26102863)
Supplement: Supplementary file 1 [file molecules-26-02863-s001.zip › molecules-1185201-supplementary.pdf]

Supplementary Material

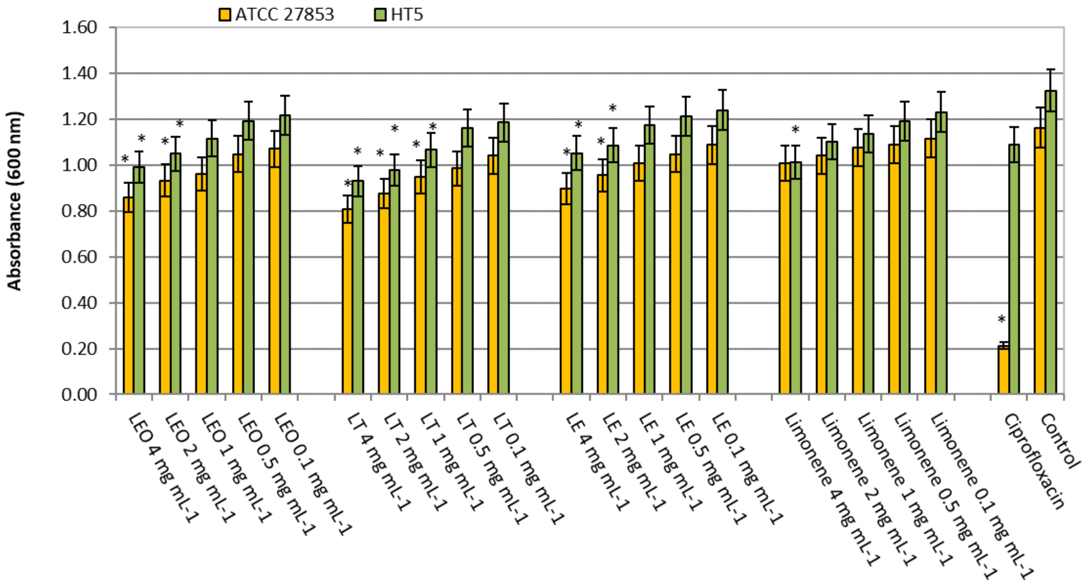

Figure S1. *Pseudomonas aeruginosa* growth without (control) and in the presence of ciprofloxacin (5 μg mL-1) or different lemon oils concentrations (0.1-4 mg mL-1). LEO: Lemon Essential Oil, LF: Lemon Terpenes, LE: Lemon Essence. Asterisk indicates significant differences compared to the respective control (Tukey`s multiple range test, P < 0.05).
